# Acute Severe Mitral Regurgitation Following Blunt Chest Trauma and a Febrile Illness in a Young Adult With Degenerative Mitral Valve Disease: A Rare Case Report

**DOI:** 10.7759/cureus.115061

**Published:** 2026-08-24

**Authors:** Wendy Saliba, Aoumar G Chamma, Linda Chamma

**Affiliations:** 1 Cardiology, University of Balamand, Beirut, LBN; 2 Urology, Lebanese University, Beirut, LBN

**Keywords:** acute mitral regurgitation, blunt chest trauma, chordae tendineae rupture, mitral valve repair, myxomatous degeneration

## Abstract

Acute severe mitral regurgitation (MR) in young adults is rare and is usually associated with infective endocarditis, trauma, congenital abnormalities, or connective tissue disorders. However, acute presentation of degenerative mitral valve disease is uncommon in young adults.

A 23-year-old man developed acute pulmonary edema following blunt precordial trauma during a football match, with a brief febrile illness occurring several days later. Transthoracic echocardiography demonstrated acute severe eccentric MR due to flail of the P2 scallop resulting from the rupture of the chordae tendineae. Comprehensive evaluation, including blood cultures, infectious workup, cardiac magnetic resonance imaging, and histopathological analysis, excluded infective endocarditis and myocarditis. The patient underwent urgent mitral valve repair with triangular P2 resection, neochordal reconstruction, and annuloplasty ring implantation. Histopathology revealed underlying myxomatous degeneration.

This case raises the possibility of a “two-hit” mechanism in which pre-existing myxomatous degeneration created structural vulnerability and the subsequent blunt chest trauma occurred in temporal proximity to chordal rupture. The accompanying febrile inflammatory state provides a hypothesis-generating biological context, but its contribution to chordal weakening or rupture cannot be established from this case.

## Introduction

Mitral regurgitation (MR) is one of the most common valvular heart diseases worldwide. In developed countries, degenerative mitral valve disease is the leading cause of primary MR and is usually observed in middle-aged or older adults [1]. The disease is characterized by myxomatous changes, leaflet redundancy, annular dilation, and progressive weakening or elongation of the chordae tendineae [2]. Although degenerative MR often evolves gradually, chordal rupture can suddenly convert a compensated lesion into acute severe MR with pulmonary edema and hemodynamic instability [3].

Acute severe MR in young adults is rare. In this age group, common etiologies include infective endocarditis, blunt or penetrating trauma, congenital abnormalities, and connective tissue disorders such as Marfan syndrome and Ehlers-Danlos syndrome [4,5]. Blunt chest trauma is a rare cause of mitral valve injury that may involve the papillary muscles, leaflets, or chordae tendineae. Diagnosis can be delayed, as early clinical manifestations are often non-specific and may be attributed to pulmonary contusion, musculoskeletal injury, or infection [6,7]. Traumatic mitral apparatus injury should be distinguished from spontaneous chordal rupture occurring in degenerative mitral valve disease. Traumatic MR results from mechanical disruption of the papillary muscles, chordae tendineae, or leaflets following external force, whereas spontaneous chordal rupture typically reflects failure of structurally weakened chordae in an underlying degenerative valve and may occur in the absence of trauma. In the present case, histopathologically confirmed myxomatous degeneration provides direct evidence of an underlying structural substrate, whereas the contribution of the preceding clinical events to the timing of chordal rupture remains uncertain.

Experimental studies have suggested that inflammatory pathways may participate in extracellular matrix remodeling in myxomatous mitral valve disease. In particular, the IL-33/ST2 pathway has been associated with valvular interstitial cell activation and changes in extracellular matrix homeostasis [8,9]. These findings provide biological plausibility for an interaction between inflammation and degenerative valve remodeling but do not establish that a brief systemic inflammatory illness can acutely alter chordal mechanical properties or cause chordal rupture in humans. We report a case of acute severe MR in a 23-year-old man with histologically confirmed myxomatous degeneration occurring in temporal association with blunt precordial trauma and a transient febrile illness.

## Case presentation

A 23-year-old man with no known cardiac history presented to the emergency department with rapidly progressive dyspnea, orthopnea, and reduced exercise tolerance over 48 hours. Seven days before presentation, he sustained a direct blunt impact to the anterior chest while playing football after colliding with another player’s shoulder. He reported immediate precordial pain and mild chest tenderness on palpation but did not seek any medical attention at the time since the symptoms improved within hours. There was no history of syncope, palpitations, or a previous cardiac murmur.

Three days after the trauma, he developed a fever reaching 38.6°C, accompanied by fatigue, myalgia, sore throat, and dry cough. He denied recent dental procedures, intravenous drug use, skin infections, recent surgery, animal exposure, consumption of unpasteurized dairy products, or a history of rheumatic fever. There was no family history of sudden cardiac death, aortic disease, known connective tissue disorders, or early mitral valve surgery.

On presentation to the emergency department, the patient appeared dyspneic and anxious. His blood pressure was 96/58 mmHg, heart rate was 122 beats/min, respiratory rate was 28 breaths/min, oxygen saturation was 88% on room air, and temperature was 37.9°C. Chest examination revealed bibasilar crackles. Cardiac auscultation showed a new grade 4/6 holosystolic murmur best heard at the apex radiating to the axilla. There were no peripheral stigmata of infective endocarditis. Examination of the chest wall showed mild anterior sternal tenderness without deformity.

Laboratory investigations revealed leukocytosis (13.2 × 10⁹/L), elevated C-reactive protein (74 mg/L), and low procalcitonin (0.08 ng/mL). High-sensitivity troponin I was mildly elevated at 86 ng/L, with a repeat value of 92 ng/L after three hours (institutional upper reference limit 34 ng/L). The minimal increase without a significant dynamic change was not suggestive of acute coronary syndrome. N-terminal pro-B-type natriuretic peptide was elevated at 1,820 pg/mL. Renal and liver function tests were within normal limits.

Given the recent febrile illness, a focused infectious work-up was performed. Three sets of blood cultures obtained before antibiotic administration remained negative. Rapid streptococcal antigen testing and throat culture were negative. Nasopharyngeal viral polymerase chain reaction testing, including influenza A and B, respiratory syncytial virus, and SARS-CoV-2, was negative. Serological testing for Epstein-Barr virus and cytomegalovirus indicated past exposure without evidence of acute infection. Urinalysis and urine culture were unremarkable. C-reactive protein decreased from 74 mg/L to 32 mg/L within the first 72 hours, and the fever resolved without the identification of a clear focus.

Chest radiography showed bilateral perihilar alveolar opacities consistent with pulmonary edema. Computed tomography of the chest showed no rib or sternal fractures, pneumothorax, or pulmonary contusion and confirmed pulmonary edema with small bilateral pleural effusions. Electrocardiography showed sinus tachycardia without ischemic ST-segment changes.

**Figure 1:**
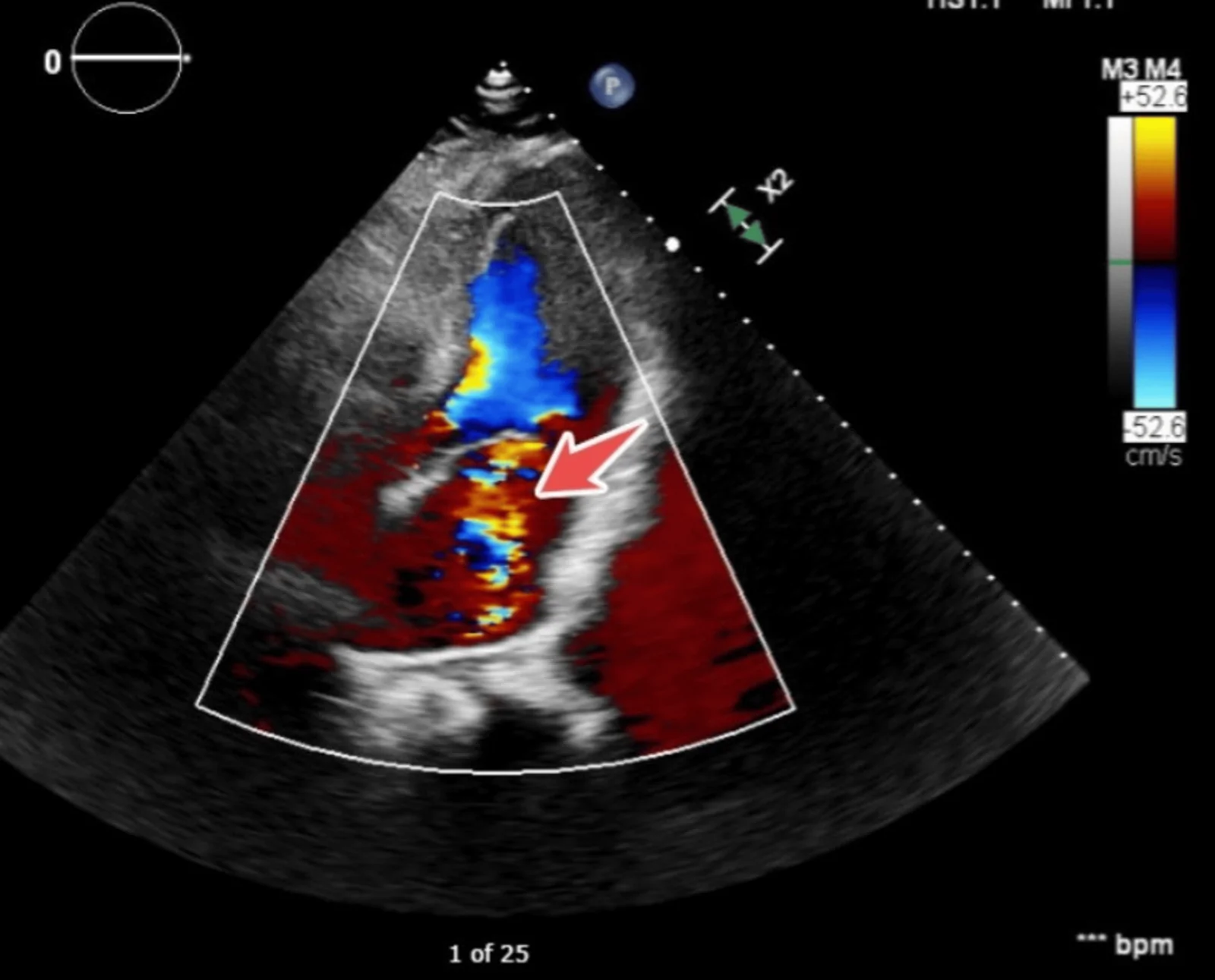
Severe eccentric anteriorly directed mitral regurgitation on color doppler echocardiography (arrow indicates the regurgitant jet)

Transthoracic echocardiography demonstrated a non-dilated left ventricle with preserved systolic function. The left ventricular end-diastolic diameter was 51 mm, end-systolic diameter 32 mm, and ejection fraction 64%. The left atrium was mildly dilated, with a left atrial volume index of 38 mL/m². There was severe eccentric anteriorly directed MR, with systolic flow reversal in the pulmonary veins (Figure 1).

Quantitative assessment showed a vena contracta width of 8 mm, an effective regurgitant orifice area of 0.48 cm² by proximal isovelocity surface area, and a regurgitant volume of 72 mL. The estimated systolic pulmonary artery pressure was 58 mmHg. Right ventricular size and systolic function were normal.

**Figure 2:**
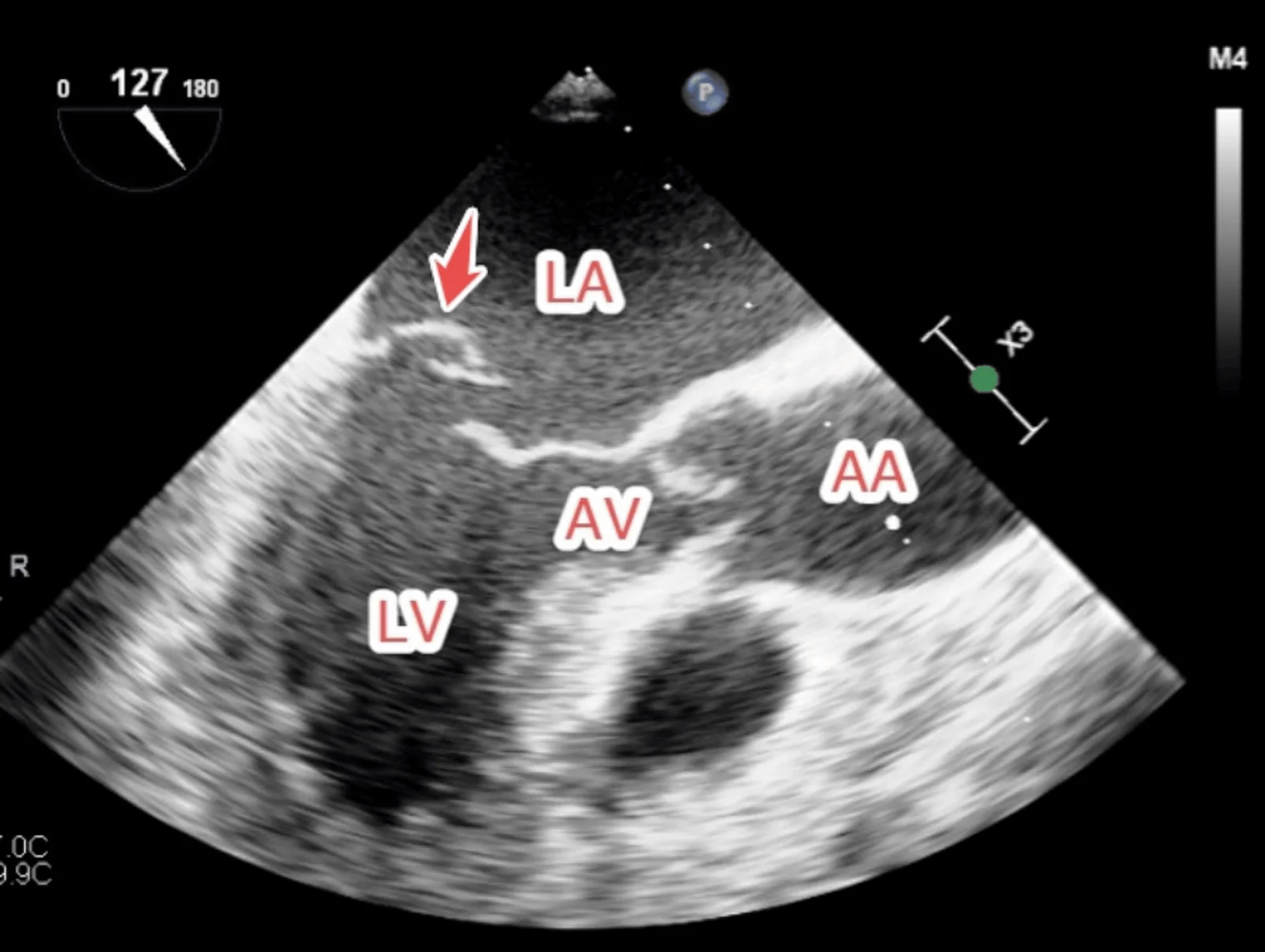
Transesophageal echocardiographic demonstration of P2 posterior mitral leaflet flail due to primary chordae tendineae rupture. The arrow highlights the flail P2 segment resulting from ruptured primary chordae tendineae. AA: ascending aorta; AV: aortic valve; LA: left atrium; LV: left ventricle.

**Figure 3:**
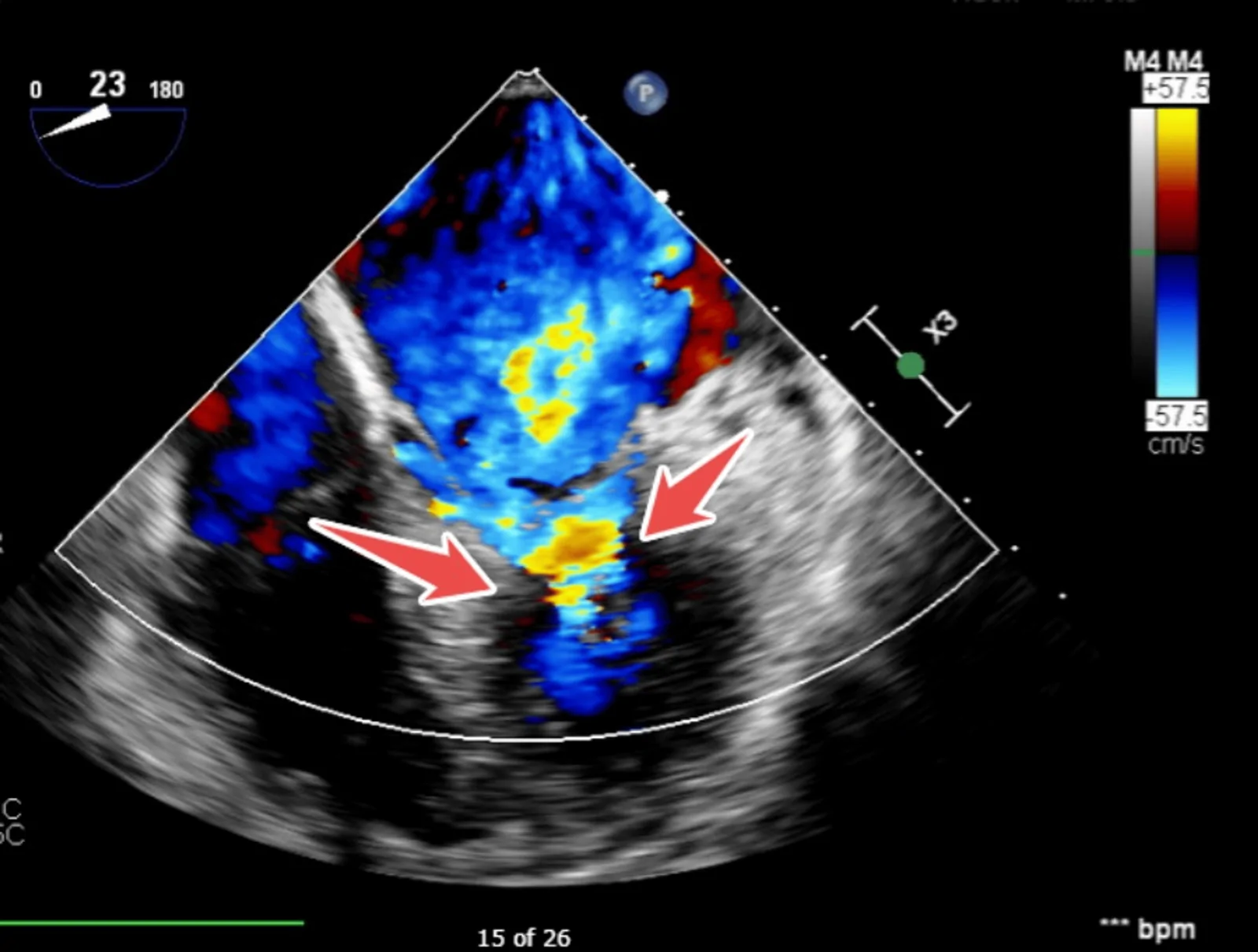
Transesophageal echocardiographic assessment demonstrating severe mitral regurgitation highlighted by arrow.

Transesophageal echocardiography confirmed flail of the P2 scallop of the posterior mitral leaflet due to rupture of the primary chordae tendineae (Figures 2, 3).

Transesophageal assessment was performed using two-dimensional multiplane imaging; three-dimensional echocardiography was not obtained. Flail gap and flail width were not prospectively quantified. The mitral leaflets appeared mildly thickened and redundant, consistent with underlying degenerative valve disease. The valve did not demonstrate the diffuse bileaflet or multisegment prolapse typical of classic Barlow disease, and no prolapse beyond the P2 scallop was identified. However, the valve morphology was not prospectively classified as Barlow disease or fibroelastic deficiency. Intraoperative inspection subsequently confirmed that the prolapse and chordal rupture were confined to the central P2 scallop, without involvement of the anterior leaflet or other mitral scallops. No vegetations, leaflet perforation, annular abscess, or intracardiac thrombus were identified.

Regarding the mild troponin elevation and preceding febrile illness, cardiac magnetic resonance imaging was performed after initial stabilization. It showed preserved biventricular systolic function without myocardial edema, late gadolinium enhancement, or parametric mapping abnormalities suggestive of acute myocarditis. Thus, the mild troponin elevation was attributed to acute hemodynamic stress in the setting of severe MR and pulmonary edema.

A connective tissue disease assessment was performed, given the patient’s young age and the myxomatous appearance of the valve. There were no clinical features suggestive of a syndromic connective tissue disorder, including Marfanoid habitus, lens dislocation, joint hypermobility, skin hyperextensibility, scoliosis, pectus deformity, or characteristic craniofacial features. Computed tomography demonstrated normal aortic root and ascending aortic dimensions. After discharge, a multigene next-generation sequencing panel for syndromic mitral valve prolapse and hereditary aortopathy was performed, including FBN1, TGFBR1, TGFBR2, SMAD2, SMAD3, TGFB2, TGFB3, SKI, ACTA2, MYH11, MYLK, COL3A1, and FLNA. No pathogenic or likely pathogenic variants were identified.

The patient was treated with supplemental oxygen, intravenous furosemide, and cautious afterload reduction. Despite medical stabilization, he remained symptomatic with persistent tachycardia and severe MR. Following heart team discussion, urgent mitral valve repair was recommended because of acute severe primary MR with pulmonary edema and favorable anatomy for repair.

Surgery was performed via median sternotomy with cardiopulmonary bypass. Intraoperative inspection revealed myxomatous degeneration of the posterior leaflet with rupture of primary chordae involving the P2 scallop. There was no evidence of leaflet perforation, purulence, annular destruction, or macroscopic features of infective endocarditis. A limited triangular resection was chosen rather than quadrangular resection since the prolapse was confined to the central P2 scallop, allowing preservation of leaflet tissue and reducing the risk of systolic anterior motion. Repair was completed with triangular P2 resection, neochordal reconstruction using expanded polytetrafluoroethylene sutures, and implantation of a 34-mm complete semi-rigid annuloplasty ring.

Intraoperative transesophageal echocardiography showed good leaflet coaptation, a mean transmitral gradient of 3 mmHg, absence of systolic anterior motion, and trivial residual MR. The patient was weaned from cardiopulmonary bypass without the need for mechanical circulatory support.

Histopathological examination of the resected leaflet tissue demonstrated myxomatous degeneration with expansion of a proteoglycan-rich extracellular matrix and fragmentation of collagen fibers. No microorganisms, vegetations, inflammatory infiltrates, or features of active endocarditis were identified. Tissue cultures were negative.

**Figure 4:**
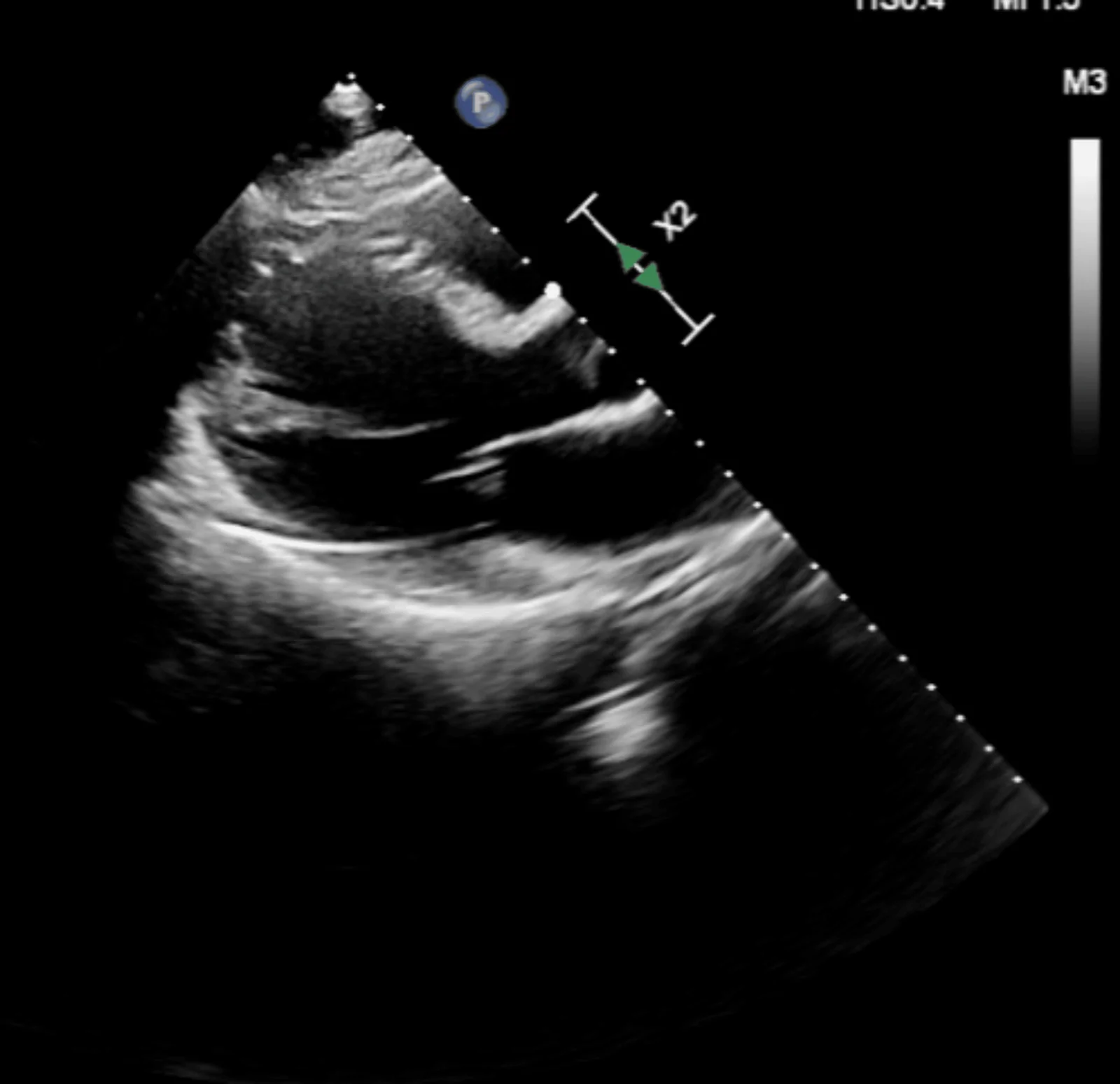
Postoperative transthoracic echocardiographic assessment following mitral valve repair

The postoperative course was uneventful. The patient was extubated on postoperative day 1 and discharged on postoperative day 7. Predischarge transthoracic echocardiography showed a left ventricular ejection fraction of 60%, trivial residual MR, a mean transmitral gradient of 3 mmHg, and an estimated systolic pulmonary artery pressure of 32 mmHg (Figure 4).

At 12-month follow-up, he remained asymptomatic and had returned to normal daily activities. Follow-up echocardiography demonstrated a durable repair with trivial residual MR, stable left ventricular dimensions (end-diastolic diameter 49 mm and end-systolic diameter 31 mm), a preserved ejection fraction of 62%, a reduced left atrial volume index (31 mL/m²), and an improved systolic pulmonary artery pressure (28 mmHg).

## Discussion

This case describes an uncommon presentation of acute severe MR in a 23-year-old man with histologically confirmed degenerative mitral valve disease. The unusual feature is the occurrence of acute chordal rupture in a young adult with a previously silent myxomatous mitral valve, in temporal association with direct precordial trauma and a short febrile inflammatory illness.

Degenerative MR is typically a chronic condition, most commonly arising from mitral valve prolapse secondary to myxomatous degeneration of the leaflets and chordae with gradual progression over time [1,2]. Acute clinical deterioration may occur when chordal rupture leads to sudden loss of leaflet coaptation. In such cases, the left atrium and pulmonary circulation do not have time to adapt, leading to acute pulmonary edema despite preserved left ventricular dimensions and systolic function [3]. The described pattern was observed in our patient, who presented with severe symptoms, a non-dilated left ventricle, and a preserved ejection fraction.

The first challenge was exclusion of infective endocarditis. Given the fever preceding acute MR, infective endocarditis was a principal differential diagnosis, particularly in a young patient with new-onset severe valvular regurgitation. However, the absence of vegetations on transesophageal echocardiography, persistently negative blood cultures, negative tissue cultures, and the absence of microorganisms or inflammatory destruction on histopathology collectively argued strongly against an infectious etiology. Current guidelines emphasize the role of integrated microbiological testing, echocardiography, and multimodality imaging in such clinical settings [10]. In this case, the combined microbiological, imaging, surgical, and pathological findings strongly supported a non-infective mechanism of chordal rupture.

The second challenge was to determine whether the blunt chest trauma could account for the valvular injury. Traumatic MR is rare, and isolated injury to the mitral apparatus is considerably less common than myocardial contusion [6]. Proposed mechanisms include sudden compression of the heart between the sternum and spine, abrupt increases in intracardiac pressure during isovolumetric contraction, and direct shearing forces involving the papillary muscles or chordae tendineae [6,7]. Although the direct precordial impact was clinically relevant, the absence of rib or sternal fractures, pneumothorax, or pulmonary contusion suggests that this was not a high-energy thoracic crush injury. Histologically confirmed myxomatous degeneration provides a clear substrate for spontaneous chordal failure. The temporal relationship with trauma raises the possibility that mechanical stress contributed to rupture, but a causal relationship cannot be established from this case.

The third consideration was whether the febrile illness contributed to the acute presentation. Viral myocarditis can present with fever, dyspnea, and troponin elevation, but cardiac magnetic resonance imaging showed no myocardial edema, late gadolinium enhancement, or other findings supporting acute myocarditis [11,12]. The infectious evaluation was also unrevealing, and the fever resolved spontaneously. The episode was therefore interpreted as a transient systemic inflammatory state. Importantly, however, there was no direct evidence that this inflammatory state altered the mechanical properties of the mitral chordae or contributed to their rupture.

The proposed “two-hit” mechanism should be regarded as a hypothesis-generating framework rather than an established causal pathway. Histopathologically confirmed myxomatous degeneration provides direct evidence that the mitral valve and chordal apparatus were structurally vulnerable. By contrast, the proposed contribution of mechanical stress from the blunt chest trauma and systemic inflammation associated with the febrile illness is inferred primarily from their temporal relationship to the clinical presentation. Experimental studies have shown that activation of the IL-33/ST2 pathway may promote valvular interstitial cell activation, endothelial-to-mesenchymal transition, proteoglycan synthesis, and extracellular matrix remodeling in myxomatous valve disease [8,9]. These findings provide biological plausibility but do not demonstrate that systemic inflammation acutely weakened the chordae in this patient or contributed causally to their rupture. Therefore, spontaneous rupture of degeneratively weakened chordae remains a plausible alternative explanation, with trauma and inflammation representing possible, but unproven, contributing factors.

This case also raises the question of whether earlier echocardiographic evaluation might have identified a pre-existing mitral valve prolapse. However, in the absence of symptoms, a murmur, or a relevant family history, routine echocardiographic screening would not generally have been indicated. Nevertheless, the case supports a low threshold for echocardiography after a blunt precordial trauma when patients present with dyspnea, pulmonary edema, a new murmur, or unexplained fever. Traumatic mitral injury may be overlooked when initial symptoms are attributed to respiratory infection, musculoskeletal injury, or pulmonary contusion [6,7].

Connective tissue disease remains an important differential diagnosis to be considered in this context, given that degenerative changes at such a young age are uncommon. In this patient, there were no phenotypic features of Marfan syndrome, Ehlers-Danlos syndrome, MASS phenotype, Loeys-Dietz syndrome, or aortopathy, and no significant family history. Genetic testing did not identify a pathogenic or likely pathogenic variant associated with syndromic mitral valve disease or aortopathy. Nevertheless, this does not exclude an underlying genetic predisposition, as early-onset myxomatous degeneration may occur outside classical syndromic presentations [5].

Surgical intervention was indicated because of symptomatic acute severe primary MR with pulmonary edema and favorable valve anatomy for repair. Current guidelines recommend mitral repair over replacement in degenerative MR when a durable repair is feasible [3,4]. Long-term data revealed improved survival and reduced valve-related complications with repair compared to replacement in appropriately selected patients [13]. Durable outcomes with low reoperation rates have been reported in large surgical series of leaflet prolapse repair [14]. Early intervention has also been reported with beneficial outcomes in patients with flail leaflet anatomy to prevent irreversible ventricular and atrial remodeling [15]. In this case, a repair strategy was tailored to address the localized P2 lesion. Limited triangular resection preserved leaflet tissue, while neochordal reconstruction restored leaflet support and annuloplasty ring implantation provided annular stabilization. The trivial residual MR and low transmitral gradient at 12-month follow-up support a durable early surgical outcome.

This case reflects the acute presentation of previously silent degenerative mitral valve disease in temporal association with blunt chest trauma and a transient febrile inflammatory state. The findings raise the possibility that mechanical stress and systemic inflammation may interact with a structurally vulnerable valve, but the relative contribution of these factors and any causal relationship cannot be established from a single case.

## Conclusions

Acute severe MR in a young adult should prompt urgent evaluation for infective endocarditis, trauma-related valve injury, myocarditis, connective tissue disease, and underlying degenerative mitral valve pathology. This case demonstrates that myxomatous mitral valve disease can rarely present with acute chordal rupture at a young age. Blunt precordial trauma and a transient febrile inflammatory illness occurred in temporal association with the acute event, but their causal contribution to chordal rupture cannot be established. Early echocardiographic assessment, careful exclusion of competing etiologies, genetic evaluation when appropriate, and prompt mitral valve repair can lead to excellent outcomes.
